# Supplementary material for: Consistent survival in consecutive cases of life-supporting porcine kidney xenotransplantation using 10GE source pigs
Source: Nat Commun. 2024 Apr 18;15:3361. doi: 10.1038/s41467-024-47679-6 (PMC11026402; doi:10.1038/s41467-024-47679-6)

Supplementary Information

Supplemental
Figure 1

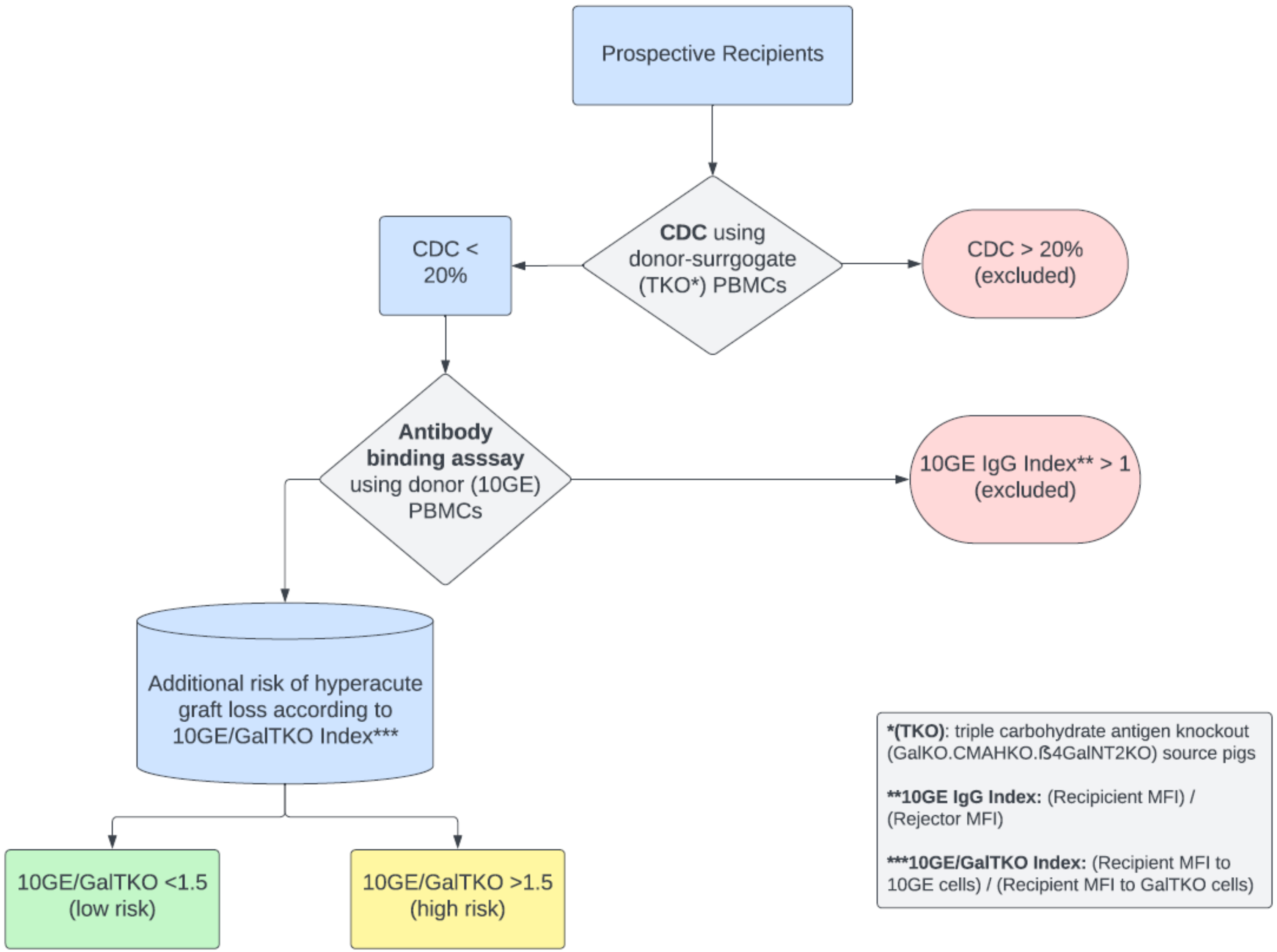

Flowchart outlining recipient selection for 10GE recipients. This two-step screening methodology incorporates donor-surrogate (TKO) and donor-specific (10GE) testing and was developed by The Yamada Lab at Johns Hopkins University.

Supplemental
Figure 2

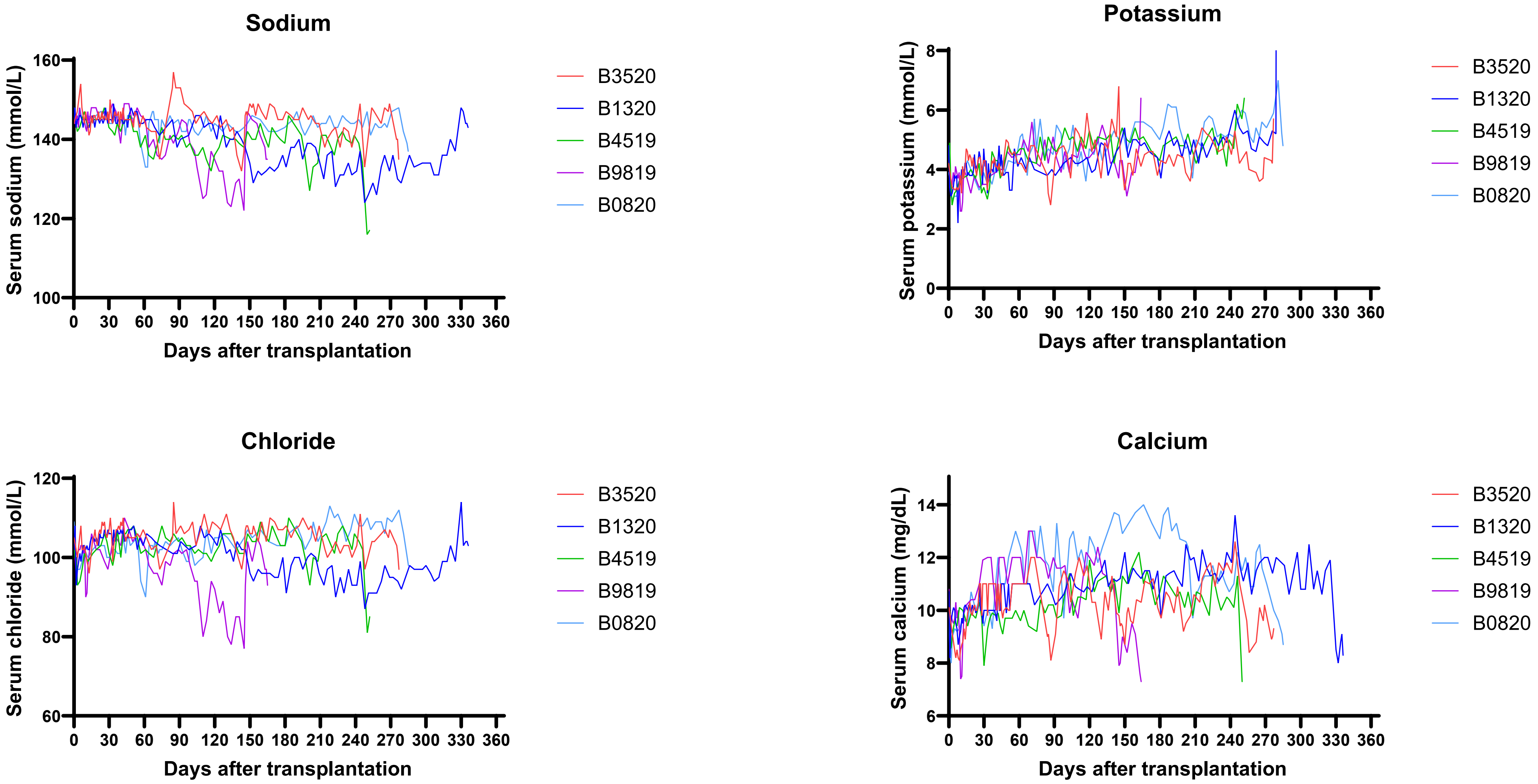

Xenograft kidneys maintained normal concentrations of sodium, potassium, and chloride. B9819 developed hyponatremia and hypochloremia associated with dehydration in the setting of adenovirus infection. Long-term survivors noted to have mild-moderate hypercalcemia.

Supplemental
Figure 3

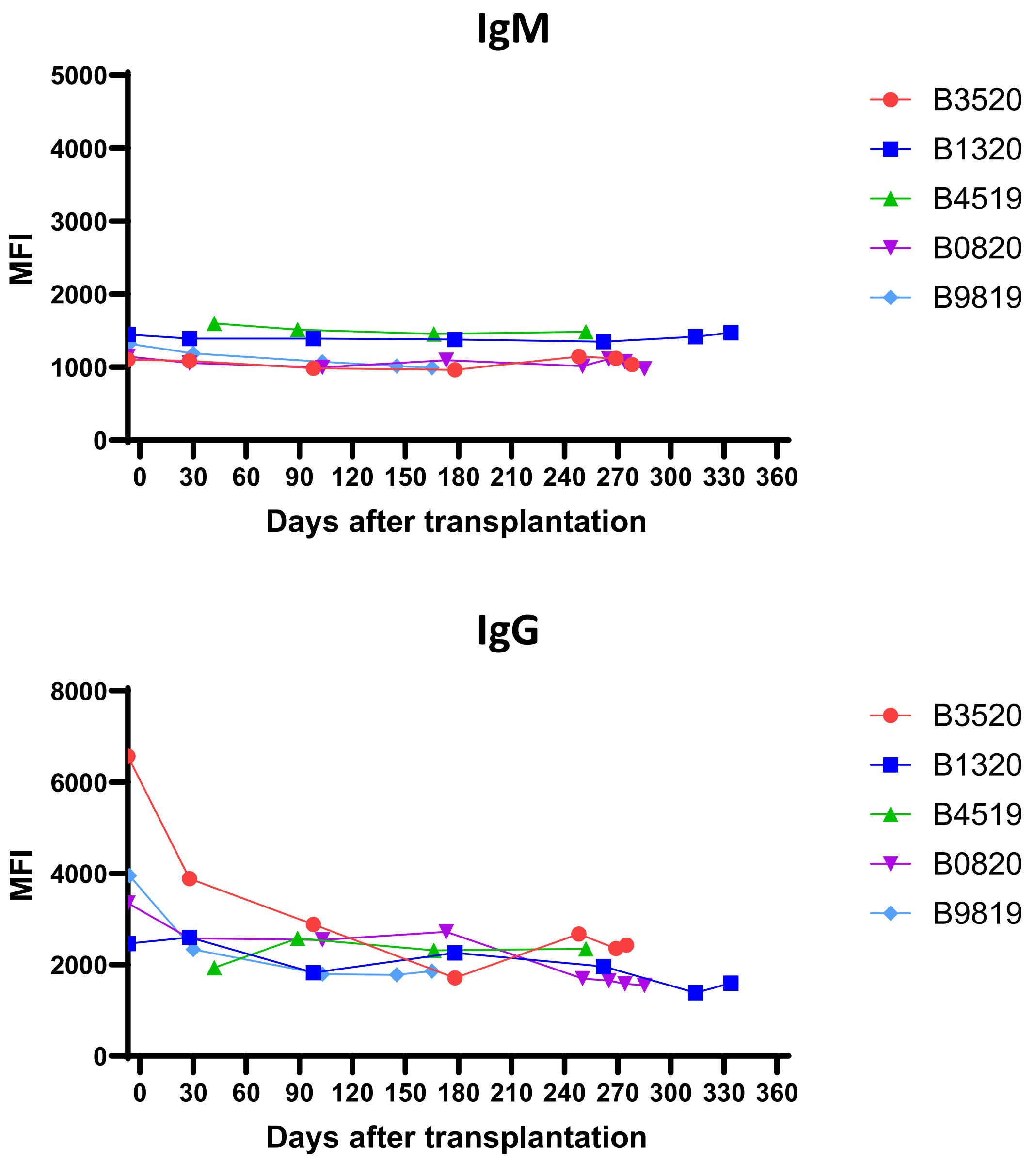

None of the recipients (including B9819 and B4519) developed donor specific antibodies as assessed by flow cytometric evaluation of recipient serum antibody binding to source pig peripheral blood mononuclear cells

Supplemental
Figure 4

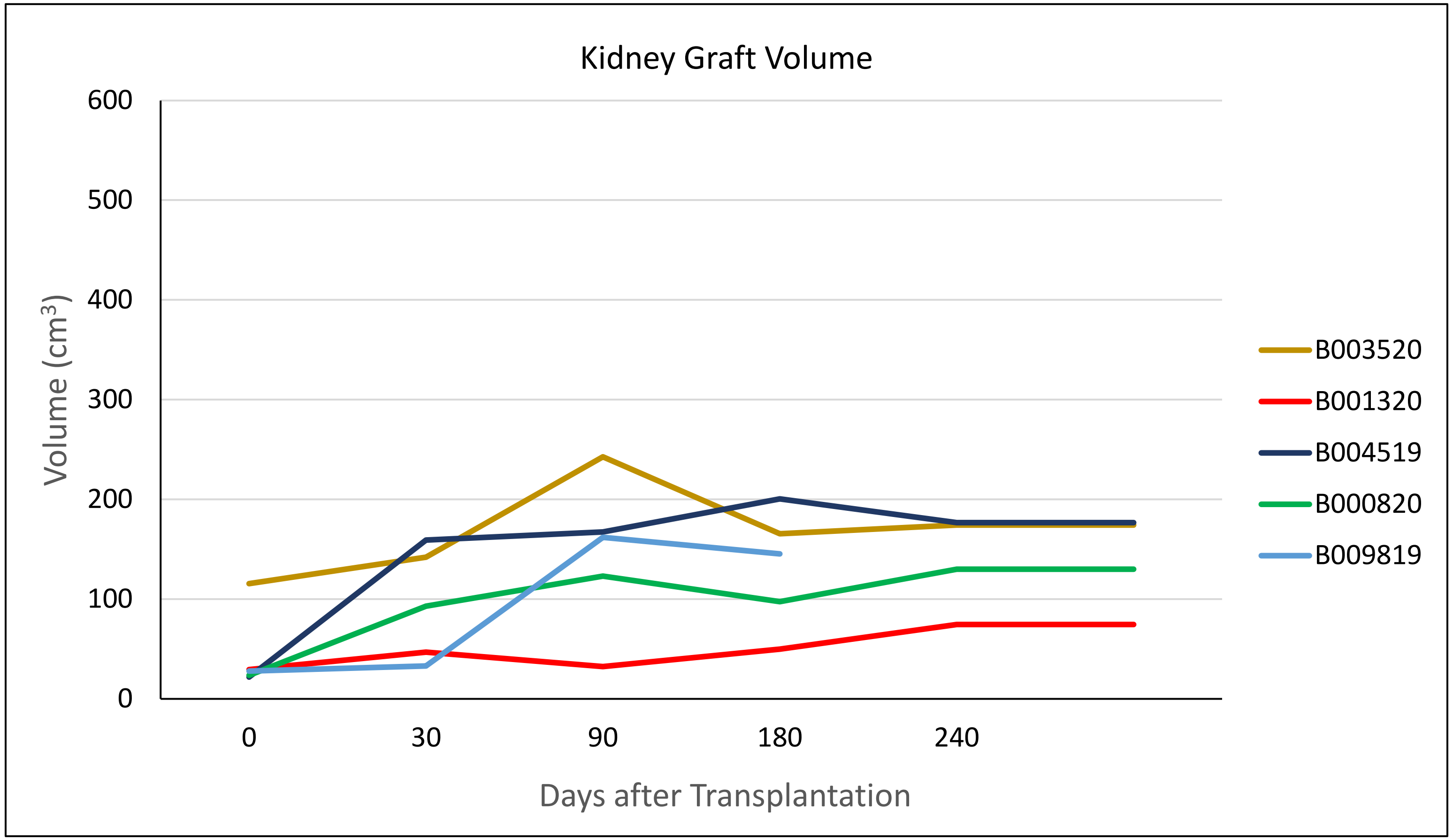

Supplement: Supplementary file 1 — Supplementary Information [file 41467_2024_47679_MOESM1_ESM.pdf]
